# Book Reviews

**Published:** 1891-02

**Authors:** 


### Book Reviews.

---

OINTMENTS AND OLEATES, ESPECIALLY IN DISEASES OF THE SKIN. By John V. Shoemaker, A. M., M. D. Professor of Materia Medica, Pharmacology, Therapeutics, and Clinical Medicine, and Clinical Professor of Diseases of the Skin in the Medico-Churugical College, of Philadelphia, etc. Second Edition. Revised and Enlarged. Philadelphia and London. F. A. Davis, Publisher, 1890.

So far as we know, the title and matter of this work are unique The use of ointments and fats is first taken up, and the reader finds many practical and interesting points here brought out. He gives the history and evolution of ointments from the time of the ancients, the relative absorbability of the different fats and oils, the effects on temperature, etc., and the injury to be done by the use of rancid fats with their free fatty acids. The author pays especial attention to lanolin, the fatty derivative from sheep's wool. Purest lanolin is given the first rank as a base for remedies desired to be absorbed.

He then gives the formulæ and uses of the official and unofficial ointments of the United States, the official ointments of British Pharmacopœiæ, and of the German, French, Austrian, Italian, Spanish, Mexican, and Chilian Pharmacopœiæ, showing a depth of research and thoroughness of work which should of itself be sufficient to recommend the book.

In conclusion he gives a detailed history of the Oleates, the process of their manufacture, their physiological action. and their therapeutic action. The author has given much attention to the Oleates, and we can find no better authority upon their preparation and use. The book would be a valuable addition to any library.

M. B. H.

TREATISE ON MASSAGE. By Douglass Graham, M. D. J. H. Vail & Co., New York, Publishers.

This edition is more comprehensive than the first, containing history of massage, the various indications for its use and method of application. The work abounds in practical observations, showing that massage has a place in mechanical therapeutics.

L. P. K.

---

TEXT-BOOK ON HYGIENE. By George H. Rohe, M. D. F. A. Davis, Philadelphia, Publisher.

Incorporated in this work are the principles upon which sanitary science is based. The type is good, the style is simple, the subjects treated are of vital importance; in fine, the work is such as to commend itself to the reader. L. P. K.

---

STRUCTURE OF CENTRAL NERVOUS SYSTEM. By Ludwig Edinger, M. D. Translated by Willis Hall Vittum, M. D., St. Paul, Minn. Edited by C. Eugene Riggs, A. M., M. D., Professor of Mental and Nervous Diseases, University of Minnesota, Member of the American Neurological Association. Published by F. A. Davis, Philadelphia. Price, $1.75 net.

This work is made up of twelve lectures delivered by the author to an audience of practicing physicians. It deals with the minute anatomy of the brain, rather than its coarse texture. This, the most difficult part of the human anatomy, is rendered as interesting as it is intricate. The book contains 133 cuts, illustrative of points under consideration. L. P. K.

